# A Rare Complication of Radiofrequency Tonsil Ablation: Horner Syndrome

**DOI:** 10.1155/2015/570520

**Published:** 2015-05-07

**Authors:** Cuneyt Kucur, Isa Ozbay, Fatih Oghan, Nadir Yildirim, Zuhal Zeybek Sivas, Sibel Canbaz Kabay

**Affiliations:** ^1^Department of Otorhinolaryngology, Dumlupinar University, 43235 Kutahya, Turkey; ^2^Department of Neurology, Dumlupinar University, 43235 Kutahya, Turkey

## Abstract

Chronic tonsillitis is a common disease, and several different surgical techniques are used to treat this condition. In recent years, techniques such as radiofrequency ablation and coblation have been commonly used for tonsil surgery. In this report, we present the cases of two pediatric patients who developed ptosis, miosis, and enophthalmos (Horner syndrome) after radiofrequency ablation for tonsil reduction and discuss the technique of radiofrequency ablation of the tonsils. In the early postoperative period, miosis and ptosis were observed on the right side in one patient and on the left side in the other patient. Both patients were treated with 1 mg/kg/day methylprednisolone, which were tapered by halving the dose every 3 days. Miosis and ptosis improved after treatment in both patients. Along with the case presentation, we discuss the effectiveness and complications of radiofrequency ablation of the tonsils. These unusual complications of tonsil ablation may help ENT physicians who do not yet have a preferred surgical technique for tonsillectomy to make an informed decision. Limited data are available about the possible complications of radiofrequency ablation of the tonsils. The present report contributes to the literature on this topic.

## 1. Introduction

Tonsillectomy has been performed since ancient times. In his book De Medici (10 BC), Cornelius Celsus described the removal of infected tonsils with the help of a finger [1]. Since the introduction of the Davis mouth opener in 1917 by Crowe Davis [2], tonsillectomy has remained one of the most frequently performed operations in children. Several different techniques and instruments are currently in use in tonsil surgery, such as, cold-knife tonsillectomy, monopolar and bipolar diathermy cryosurgery, absorbent diathermy bipolar scissors, KTP-532 laser, microscopic bipolar diathermy, and various radiofrequency (RF) ablation and coblation techniques. RF ablation involves the insertion of a probe into the tissues to destroy cells with ionizing energy. This results in tissue volume reduction within the following few days. Temperatures ranging from 40°C to 70°C are attained in the tissues, and the desired amount of energy can be transferred to the tissues. RF ablation is a successful and reliable technique [3, 4].

In this report, we present the cases of two pediatric patients who developed ptosis, miosis, and enophthalmos after RF ablation of the tonsils and discuss the technique of radiofrequency ablation of the tonsils. Tonsil reduction via RF ablation has been shown to be superior to conventional tonsillectomy in terms of postoperative pain (duration of use of narcotic pain medications) and recovery (resumption of daily activities and a normal diet) [3, 4]. However, reports on the complications of RF tonsil reduction are inconclusive. The present case report will contribute to the literature on this topic.

## 2. Case

**Figure 1:**
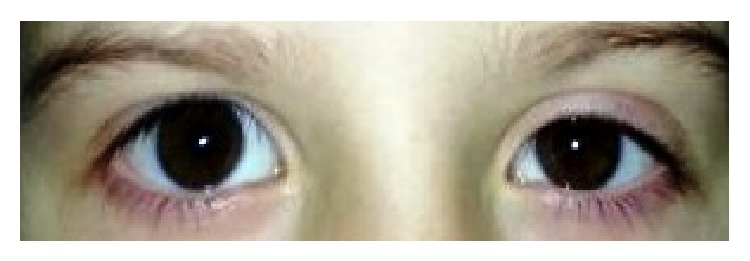
A 5-year-old boy with miosis and ptosis.

Two 5-year-old boys with witnessed sleep apnea were admitted to our clinic in 2006 and 2013. Adenoid and tonsillar hypertrophy were detected on endoscopic examination, and the patients underwent adenoidectomy and RF tonsil ablation. Bilateral ablation of the tonsillar tissue was achieved with monopolar RF probes with 15 volts (Pellevé, Ellman International, Inc., Oceanside, NY) inserted at six different sites. In the early postoperative period, ptosis, miosis, and enophthalmos were observed on the right side in the first patient and the left side in the second patient (Figure 1).

**Figure 2:**
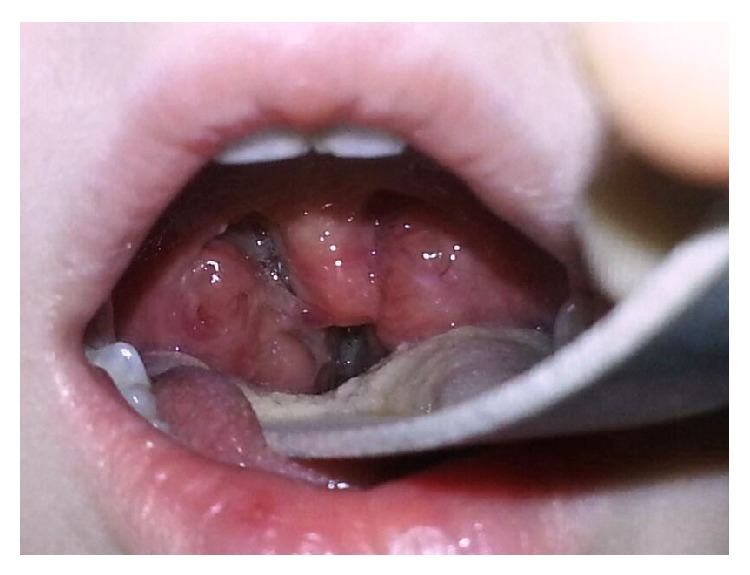
Postoperative view of tonsillar hypertrophy.

Magnetic resonance imaging of the pharynx did not show any positive findings other than bilateral tonsillar hypertrophy which is also obvious in oropharyngeal examination (Figure 2). Neurological examination revealed unilateral anhidrosis, miosis, ptosis, and enophthalmos. A diagnosis of Horner syndrome was considered. Both patients were treated with high-dose methylprednisolone (1 mg/kg/day), which was tapered by halving the dose every third day. The postoperative symptoms improved 2 months after the treatment in both patients, but mild ptosis could be observed at 1 year after the surgery in one patient and at 7 years after the surgery in the other patient.

## 3. Discussion

Over the last 10 years, surgeons have recognized the advantages of minimizing injury to the tissues surrounding the tonsillar fossa and have described procedures to preserve the overlying mucosa to avoid or lessen the problems associated with complete tonsillectomy. Numerous publications are available on RF tonsil ablation and its success [3–6]. However, because of questions about the effectiveness of this treatment [7], some surgeons do not prefer this technique.

Nelson determined that the tonsil volume shrinks by 30%–70% after RF ablation and that this technique is superior to cold-knife tonsillectomy in terms of postoperative pain and complications [3, 4]. Coticchia et al. [5] compared the safety and efficacy of RF tonsil ablation and blunt-dissection tonsillectomy in patients with obstructive sleep apnea caused by hypertrophic tonsils. They found no difference in treatment outcomes in both groups. However, in terms of postoperative pain and weight loss, RF ablation was superior to blunt dissection tonsillectomy [5].

Friedman et al. [7] examined 150 patients treated with three different methods of tonsillectomy: ablation (50 patients), coblation (50 patients), and cold knife (50 patients). The following outcomes were compared among the three groups: level of postoperative pain, time until resumption of daily activities, time until resumption of a normal diet, and requirement of narcotic analgesics. The authors concluded that the most advantageous technique was the coblation method. Although the ablation technique was associated with low complication and morbidity rates, tonsillar tissue persisted in 70% of patients. Thus, the ablation technique was not effective [7]. Furthermore, postoperative edema after RF ablation can lead to respiratory obstruction [4].

The results of the above studies indicate that tonsillectomy is more effective than tonsil reduction in preventing recurrence. Considering the postoperative complications, the RF ablation is more successful than the cold-knife approach [3–6].

Numerous studies in literature mentioning about post-tonsillectomy Horner's syndrome [8, 9]. In most of the cases symptoms were temporary and authors postulated that Horner's syndrome occurred by a direct action of the local anesthesia on components of the sympathetic chain. Permanent Horner's syndrome after tonsillectomy has been reported rarely [10]. In the presenting cases, local anesthetic drugs were not used and the complication occurred after RF tonsil ablation.

In our patients, Horner syndrome developed after tonsil reduction via RF ablation at high voltage (15 volts). The etiology of ptosis and miosis in our patients was not fully understood, and we propose two different theories to explain the underlying causes. The first reason could be pressure on the sympathetic plexus due to emerging inflammation and edema around the tonsillar tissue after RF ablation. The second reason could be direct damage to the sympathetic plexus because of the high temperatures used. Both patients responded well to the steroid treatment. However, our experience illustrates that the use of RF ablation of the tonsils must be reconsidered and surgeons should be careful where they put hot devices in the depths of the tonsil.

In 2000, using both in vivo and in vitro studies, Nelson [3] showed that RF ablation is a reliable technique. Furthermore, he compared probes with one head, two heads, and four heads and found that the two-headed probe was easier to use and heated tissue only up to the tonsillar capsule, and not beyond the capsule [3]. In a 2003 study, Nelson reported that, with probes equipped with heat protection, it was safe to operate on six different points with a minimum of 10 volts and a maximum of 15 volts [4].

In a series of 150 tonsillectomy operations, Friedman et al. used 6000–8000 J of energy to ablate the tonsils at 6–8 different points with bipolar radiofrequency probes equipped with heat protection [7]. However, in the current presentation, such a complication occurred when RF ablation (15 volts) was performed with a monopolar probe. This indicates that bipolar radiofrequency probes with heat protection should be used in tonsil treatments. The complication may also be attributable to the use of 15 volts of energy. Therefore, the use of high-energy ablation for tonsillectomy must be reconsidered.

## 4. Conclusion

Many techniques are used for tonsil surgery. In the current report, we have discussed the technique of RF ablation of the tonsils. RF ablation is superior to cold-knife tonsillectomy in terms of postoperative complications. However, the effectiveness of the radiofrequency method must be reexamined, and surgeons must have more control over the surgical steps.
